# Firearm Storage in US Households With Children: Findings From the 2021 National Firearm Survey

**DOI:** 10.1001/jamanetworkopen.2021.48823

**Published:** 2022-02-22

**Authors:** Matthew Miller, Deborah Azrael

**Affiliations:** 1 Bouvé College of Health Sciences, Department of Health Sciences, Northeastern University, Boston, Massachusetts; 2 Harvard Injury Control Research Center, Harvard T.H. Chan School of Public Health, Boston, Massachusetts

## Abstract

This survey study uses responses from the 2021 National Firearms Survey to analyze firearm storage practices in US households with children.

## Introduction

In 2015, one-third of all households with children contained firearms, 21% of which contained at least 1 firearm that was both loaded and unlocked. As a result, approximately 4.6 million children lived in a home with loaded and unlocked firearms.^1^ The nationally representative survey study reported herein updates these estimates as of April 2021, 12 months into an unprecedented and sustained surge in firearm purchases.^2,3^

## Methods

Data are from a nationally representative survey of US adults conducted April 15 through 26, 2021, with respondents drawn from an online sampling frame of 55 000 US adults recruited using address-based sampling. A description of the survey methods is available elsewhere.^3^ The institutional review boards at Northeastern University, Boston, Massachusetts, and Harvard’s School of Public Health, Boston, determined the survey did not require review because the deidentified data did not qualify as human participants research. We followed the American Association for Public Opinion Research (AAPOR) reporting guideline for survey studies.

Panel members were screened with questions about household firearm ownership, including whether they personally owned firearms. Panel members were asked (1) Do you personally own a working gun (yes/no)? and (2) Does anyone else in your household own a working gun (yes/no/don’t know)? Firearm owners were asked (1) Do you store any of the guns you keep in or around your home loaded (yes/no)? (2) Do you store any of the guns you keep in or around your home unlocked (yes/no)? and, if they answered yes to both questions, (3) Do you store any of the guns you keep in or around your home both loaded and unlocked (yes/no)? Race and ethnicity were self-reported, using profile categories supplied by the survey firm (Ipsos).

Poststratification weights were applied to adjust for nonresponse and undercoverage or overcoverage from the study-specific sample design relative to expected distributions from the US Census Current Population Survey and the American Community Survey. Analyses used the SVY suite of commands in Stata, version16 (StataCorp LLC) to produce prevalence estimates with 95% CIs.

## Results

Of the 29 985 adult panel members invited to participate, 19 049 (63.5%) did so. Among all respondents, 9144 (48.0%) were men, 9905 (52.0%) were women, 62.0% were White non-Hispanic, and 33.3% lived in households with children younger than 18 years. Of the adults with children, 40.4% (95% CI, 38.6%-42.2%) who responded to the survey lived in a household with firearms. Of these, 29.3% (95%, CI, 27.7%-30.9%) owned firearms, and 11.1% (95% CI, 9.9%-12.4%) lived in a home with firearms but did not personally own a firearm (Table). The mean number of children per firearm-owning household with children (1.9; 95% CI, 1.9-2.0) was comparable to that in households with children and no firearms (1.8).

**Table.** Characteristics and Storage Practices of Firearm Owners With Children^a^

Respondent characteristic	Firearm owners with children in the household, weighted % (95% CI) (n = 1363)
Age group, y	
18-29	13.0 (10.4-16.2)
30-44	51.9 (48.7-55.1)
45-59	27.4 (24.9-30.2)
≥60	7.7 (6.4-9.2)
Sex	
Female	39.8 (36.7-43.0)
Male	60.2 (57.0-63.3)
Race and ethnicity^b^	
Hispanic	14.9 (12.6-17.6)
Non-Hispanic	
Black	11.0 (8.9-13.4)
White	68.2 (65.0-71.3)
Other	5.9 (4.5-7.7)
Marital status	
Married	77.0 (74.1-79.8)
Widowed	2.0 (1.4-2.9)
Divorced or separated	8.2 (6.7-9.9)
Never married	12.8 (10.4-15.6)
Educational level	
Less than high school	6.5 (4.5-9.3)
High school	25.2 (22.3-28.4)
Some college	36.1 (33.2-39.2)
Bachelor's degree or higher	32.2 (29.6-34.9)
Metropolitan Statistical Area Residency	81.2 (78.6-83.6)
Region	
Northeast	8.8 (7.3-10.7)
Midwest	24.9 (22.3-27.6)
South	43.6 (40.4-46.8)
West	22.7 (20.2-25.6)
Rurality	
Urban	26.2 (23.5-29.2)
Rural	27.4 (24.6-30.4)
Suburban	46.4 (43.2-49.6)
Annual income, $	
<25 000	6.8 (5.2-8.8)
25 000-49 999	14.7 (12.4-17.4)
50 000-99 999	34.5 (31.5-37.6)
≥100 000	44.0 (40.9-47.2)
Type of firearm owned	
Handguns only	28.9 (24.8-33.4)
Long guns only	16.9 (13.7-20.6)
Handguns and long guns	54.2 (49.7-58.7)
Storage practices	
Loaded and unlocked	15.0 (12.3-18.2)
Loaded and locked, or unloaded and unlocked	40.9 (36.6-45.3)
Unloaded and locked	44.1 (39.8-48.5)
Unlocked	36.1 (32.0-40.4)
Loaded	37.1 (33.1-41.4)

^a^ Altogether, 40.4% (95% CI, 38.6%-42.2%) of adults with children who responded lived in a household with firearms (as did approximately 31 million children): 29.3% (95% CI, 27.7%-30.9%) of these adults personally owned firearms and 11.1% (95% CI, 9.9%-12.4%) lived in a home with firearms but did not own any firearms themselves. The mean number of children per firearm-owning household with children (1.9) was comparable to that in households with children and no firearms (1.8).

^b^ Self-reported race and ethnicity response options included Alaska Native or American Indian, Asian, Black or African American, Cuban, Cuban American, Hispanic origin, Mexican, Mexican-American, Chicano, Native Hawaiian or Pacific Islander, Puerto Rican, White, more than 2 races, and Other Spanish, Hispanic, or Latino. In the Table, Other indicates the combination of all other race and ethnicity options that are not currently listed in the Table.

Most of the firearm owners with children (n = 1363) were male, were White, were married, lived in either a rural or suburban area, and had attended some college (Table). Of these, 36.1% (95% CI, 32.0%-40.4%) had unlocked firearms, and 37.1% (95% CI, 33.1%-41.4%) had loaded firearms. Of these, 15.0% (95% CI, 12.3%-18.2%) stored at least 1 firearm loaded and unlocked (least safe), and 44.1% (95% CI, 39.8%-48.5%) stored all firearms unloaded and locked.

## Discussion

These results indicate that in April 2021, approximately 30 million children lived in households with firearms, 7 million more than in 2015.^1,2^ Firearm owners with children were more likely to store all household firearms locked and unloaded in 2021 (44.1%) compared with 2015 (29%)^1^ and were slightly less likely to have firearms that were both loaded and unlocked (15.0% vs 21%^1^).

Nevertheless, the trend toward safer storage we observed in 2021 was offset by the increase in the proportion of adults with children who lived in households with firearms. As a result, our estimate of the number of children who lived in a household with loaded and unlocked firearms in 2021 (4.6 million) was not meaningfully different from the estimate reported in the 2015 National Firearms Survey.

Our study has limitations. This study’s results should be interpreted considering potential inaccuracies due to social desirability, with such bias likely underestimating the prevalence of unsafe storage.

The American Academy of Pediatrics recommends that all firearms in households with children should be unloaded and in locked storage, with ammunition stored separately. Our findings underscore the ongoing need for more effective efforts to reduce children’s exposure to unsafely stored firearms, especially considering recent increases in new firearm owners, including those with children.^2,3,4,5^
